# Death of Dr. Drake

**Published:** 1852-12

**Authors:** 


### THE DEATH OF DR. DRAKE.

Our readers will receive with deep sensation the intelligence of the death of Dr. Daniel Drake, long familiarly known to them as Senior Editor of this Journal. He died in Cincinnati, at 6 o'clock, on the evening of the 5th of Nov., of an affection of the brain, which he had been accustomed to regard as of a congestive character. He was just about entering upon a course of lectures in the Medical College of Ohio, when attacked by the disease which terminated his useful life.

Dr. Drake was one of the pioneers of Medicine in the West, and one of the great men of the American medical profession. He was industrious, energetic, persevering, active, observant, and original. Of all the men with whom it has been our fortune to live on terms of intimacy, our impression is that he possessed the best mind—the most acute, analytical and comprehensive. He has left behind him, though in an unfinished condition, a work which, in our opinion, ranks first as an intellectual effort among the productions of American physicians. We trust that the second volume was so far advanced that his literary executors will have no trouble in completing it.

Dr. Drake had just entered his 68th year. He visited his friends in Louisville during the late session of the Kentucky Medical Society, and was here on the return of his 67th birth-day. His health appeared to be perfect, and no one could perceive any abatement of that clearness and strength of mind and activity of body for which he was so remarkable. As he was of a long-lived family, he had the prospect of a lengthened green old age before him. It is supposed that the anxiety and effort incident to the opening of the College to which he had returned so often, and concerning the success of which he had felt so keen a solicitude, had something to do in bringing on the fatal affection of his brain. It had long been his wish, often expressed, that he might die in Cincinnati and be followed to his grave by a large body of medical students and practitioners. His wish was gratified—and his friends have the consolation, not only that he lived long and usefully, but that his last hours were cheered by the hopes of the Christian.

Dr. Drake's life was eventful beyond that of most medical men. He was at once a laborious practitioner, a prominent public teacher, and a distinguished author. Entering into the practice of physic after attending one course of lectures, he had written a work which bore his name far beyond the boundaries of his own country before he returned to the University to graduate; and he had hardly taken his degree before he became a public teacher. He was a member of the first Medical Faculty ever organized in the Valley of the Mississippi, and took part in the first course of lectures; and his name is attached to the diploma of the first doctor ever graduated in the west. He was the first medical student in Cincinnati, and the first medical author.

Quitting the *Lexington Medical School*, after a single session, he returned to Cincinnati and procured a charter for the *Medical College of Ohio*, organized it, and in the fall of 1819 commenced a course of lectures in it. In the spring, as President of the Faculty, which filled and vacated its own board, he was dismissed by his two colleagues, one making the motion and the other seconding it, he in the chair putting the question. In 1823 he was again in Lexington lecturing with wonderful animation on Materia Medica in *Transylvania University*. Two years afterwards he was translated to the chair of Theory and Practice, and after two courses of lectures on that branch resigned his professorship and returned to his practice in Cincinnati.

In 1830 he was elected Professor of Theory and Practice in the *Jefferson Medical College* of Philadelphia, where he delivered a single course of lectures, resigning in the spring. The year following he organized the *Miami Medical College*, but a union of the new school with the Ohio Medical College having been brought about, he delivered a course of lectures on Clinical Medicine in the latter institution. In the spring he resigned. A few years later, he organized the *Cincinnati Medical College*. In 1839 he was invited to the *Medical Institute of Louisville*, where he labored until the spring of 1849, when he resigned his chair. The next winter he was found in his old chair in the *Medical College of Ohio*, from which he had been so unwisely expelled thirty years before. In the spring he again resigned; in the autumn he was recalled to Louisville, and after two courses returned to the Medical College of Ohio once more, where his eventful career has closed.

It would be with us a labor of love to write an eulogy upon our departed friend, but from the subjoined proceedings of the Medical Faculty of the University of Louisville, it will be seen that this task has been committed to one whose association with the deceased was longer and more intimate than ours.

At an early hour on Saturday morning, November 6, 1852, the Dean of the Medical Faculty of the University of Louisville announced to the medical class the melancholy intelligence of the death of Dr. Daniel Drake, so long identified with the University as Professor of the the Theory and Practice of Medicine, and late Professor of the Corresponding Chair in the Medical College of Ohio. As a mark of respect to the deceased, all further public exercises at the College were suspended until Monday. At a meeting of the Faculty at 5 o'clock, P. M., the following resolutions, offered by Prof. Gross, were unanimously adopted:

Whereas, it has pleased the Almighty Disposer of Events to remove from the scene of his earthly labors and the field of his usefulness and renown, Dr. Daniel Drake, Professor of Medicine in the Medical College of Ohio, and for many years a member of the Faculty of the University of Louisville, and whereas the deceased was greatly endeared to us by his many ennobling qualities, personal, social, and professional, by the great purity and uprightness of his life, and by his extraordinary mental endowments, his profound and various learning, and his zealous and untiring industry; therefore—

*Resolved,* That we have received with unfeigned sorrow the melancholy intelligence of the death of our late friend and colleague, who for so many years held and adorned the Chair of Theory and Practice of Medicine in this University, and who contributed so much to its elevation and usefulness.

*Resolved,* That in the death of Dr. Drake the American medical profession has lost one of its brightest ornaments, medical science one of its most zealous cultivators, and medical philosophy one of its most successful interpreters.

*Resolved,* That we will cherish his memory with affectionate regard and reverence, and that we acknowledge with the liveliest gratitude the debt which our profession and country owe to his labors and services.

*Resolved,* That this Faculty tender to the family of the deceased their heartfelt sympathy, and that the Dean be requested to transmit to them a copy of these resolutions.

*Resolved,* That these proceedings be published in the daily papers of the city, and in the Western Journal of Medicine, of which the deceased was for many years a distinguished editor.

On motion of Prof. Silliman, it was further—

*Resolved,* That Prof. Gross be requested to prepare a sketch of the life and services of Dr. Drake, as a memorial of the affectionate regard of this Faculty for a great and good man, and that the same be pronounced, if practicable, on some convenient occasion during the present session of this institution, before its officers and students.

L. P. YANDELL, Dean.

Since the foregoing was written, the following "Card" has been received, from which our readers will learn that the second volume of Dr. Drake's invaluable work will appear without any avoidable delay:

A Card.—The undersigned desires to make known to the medical profession throughout the country, that the late Dr. Daniel Drake had prepared a considerable portion of the second volume of his work on the Diseases of the Interior Valley of North America; the printing of which it was his expectation to have commenced early in the ensuing year.

It is the desire and intention of his children that the portion so prepared should not be lost to the Profession. It will be immediately revised by a competent person, and put, as nearly as practicable, in a shape to make it, as far as he wroie it, available to the public. As soon as this is accomplished it will be published.

CHAS. D. DRAKE, of St. Louis.

NOVEMBER 18, 1855.

